# Family history of type 2 diabetes delays development of type 1 diabetes in TEDDY children with islet autoimmunity

**DOI:** 10.1007/s00125-025-06613-1

**Published:** 2025-12-08

**Authors:** Riitta Veijola, Roy N. Tamura, Joanna L. Clasen, Helena Elding Larsson, Katharina Warncke, Andrea K. Steck, Michael J. Haller, Berglind Jonsdottir, Beena Akolkar, William A. Hagopian, Marian J. Rewers, Jin-Xiong She, Anette-Gabriele Ziegler, Jeffrey P. Krischer, Jorma Toppari, TEDDY Study Group

**Affiliations:** 1 Department of Pediatrics, Research Unit of Clinical Medicine, Medical Research Center, University of Oulu, Oulu, Finland; 2 Department of Pediatric and Adolescent Medicine, Oulu University Hospital, Oulu, Finland; 3 Health Informatics Institute, Morsani College of Medicine, University of South Florida, Tampa, FL USA; 4 Department of Clinical Sciences Malmö, Lund University, Lund, Sweden; 5 Department of Pediatrics, Skåne University Hospital, Malmö/Lund, Sweden; 6 Institute of Diabetes Research, Helmholtz Zentrum München and Forschergruppe Diabetes, Klinikum rechts der Isar, Technische Universität München and Forschergruppe Diabetes e.V., Munich, Germany; 7 Department of Pediatrics, Klinikum Rechts der Isar, Technische Universität München, Munich, Germany; 8 Barbara Davis Center for Childhood Diabetes, University of Colorado School of Medicine, Aurora, CO USA; 9 Department of Pediatrics, University of Florida, Gainesville, FL USA; 10 Division of Diabetes, Endocrinology, & Metabolism, National Institute of Diabetes, Digestive, & Kidney Diseases, National Institutes of Health, Bethesda, MD USA; 11 Pacific Northwest Diabetes Research Institute, Seattle, WA USA; 12 Medical College of Georgia, Augusta University, Augusta, GA USA; 13 Institute of Biomedicine, Research Centre for Integrative Physiology and Pharmacology, University of Turku, Turku, Finland; 14 Centre for Population Health Research, University of Turku and Turku University Hospital, Turku, Finland; 15 InFLAMES Flagship Research Centre, University of Turku, Turku, Finland; 16 Department of Pediatrics, Turku University Hospital, Turku, Finland

**Keywords:** Autoimmune disease, Gestational diabetes, HLA, Islet autoantibodies

## Abstract

**Aims/hypothesis:**

The aetiology of type 1 diabetes remains elusive. Family history of type 1 diabetes increases the disease risk but the role of other autoimmune diseases or type 2 diabetes in the family are unclear. Here, we aimed to analyse the effect of family history of diabetes and autoimmune diseases on development of islet autoimmunity and progression to type 1 diabetes.

**Methods:**

The Environmental Determinants of Diabetes in the Young (TEDDY) study is a prospective observational cohort study of children recruited as newborns in 2004–2010 at clinical centres in Finland, Germany, Sweden and the USA. A total of 8676 children with high-risk *HLA-DR-DQ* genotype for type 1 diabetes fulfilled the eligibility criteria for regular follow-up. Questionnaire-based family history of all types of diabetes and autoimmune diseases among first- and second-degree relatives (FDRs and SDRs; data available for 8558 and 7479 children, respectively) was collected. The main outcomes were development of islet autoimmunity and progression from autoimmunity to type 1 diabetes. Data until 31 January 2016 were analysed.

**Results:**

Persistent islet autoantibodies were found in 669 children and type 1 diabetes in 233 children (45% and 46% female sex, respectively). The median follow-up time after seroconversion was 6.5 years (IQR 3.3–8.5). Having an FDR with type 1 diabetes increased the child’s risk of islet autoimmunity (HR 2.2 [95% CI 1.8, 2.8]; *p*<0.001), particularly if the father or sibling had type 1 diabetes. Islet autoimmunity was also associated with family history of type 1 diabetes in an SDR when participants having an FDR with type 1 diabetes were excluded from the analysis (HR 1.4 [95% CI 1.1, 1.8]; *p*=0.017). Notably, progression from autoantibody positivity to type 1 diabetes was significantly delayed in children having type 2 diabetes in an SDR (HR 0.61 [95% CI 0.44, 0.86]; *p*=0.004). Islet autoimmunity or progression to type 1 diabetes were not associated with other types of diabetes or autoimmune diseases in the family.

**Conclusions/interpretation:**

Family history of diabetes is differentially associated with development of islet autoimmunity and progression to type 1 diabetes. The contribution made by familial, genetic and environmental factors to the two phases of the disease pathogenesis deserves distinct analyses.

**Data availability:**

Data reported here can be obtained by request at the NIDDK Central Repository website, Resources for Research (R4R), https://repository.niddk.nih.gov/.

**Graphical Abstract:**

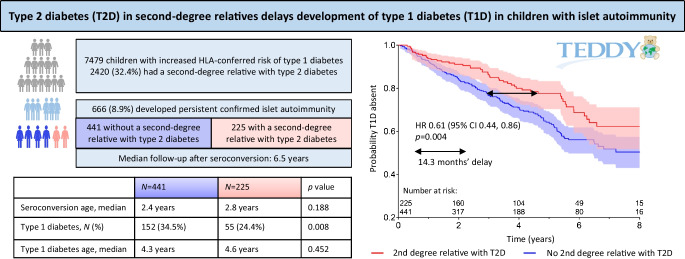

**Supplementary Information:**

The online version of this article  (10.1007/s00125-025-06613-1) contains peer-reviewed but unedited supplementary material.


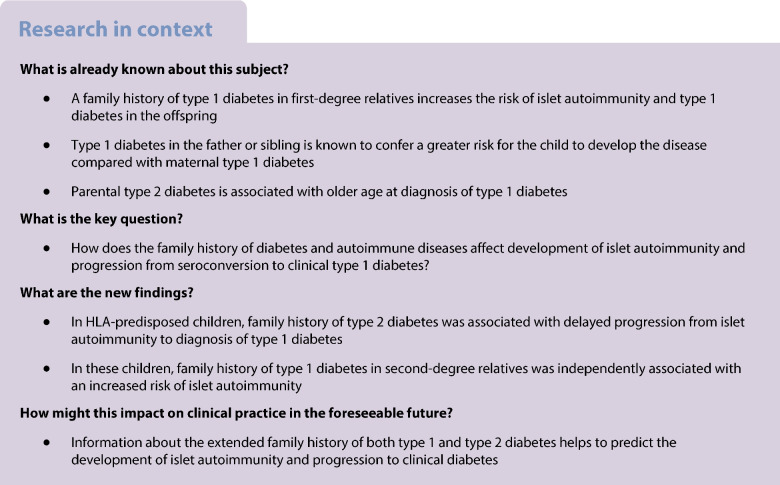


## Introduction

Type 1 diabetes is more frequent among first-degree relatives (FDRs) of children with type 1 diabetes, and also among second-degree relatives (SDRs), compared with control children [1, 2]. Other autoimmune diseases, such as thyroid autoimmunity, coeliac disease and rheumatoid arthritis, also occur more frequently among children with type 1 diabetes and their family members [2–5]. Earlier studies have compared the characteristics of type 1 diabetes at diagnosis and thereafter in children with or without a family history of type 1 diabetes or other autoimmune diseases [6, 7]. Little is known, however, about the role of family history in the initiation of the disease process (i.e. early signs of autoimmunity) or progression from autoimmunity to clinical type 1 diabetes.

Variable data have been reported about the frequency of type 2 diabetes and gestational diabetes in families with a child having type 1 diabetes. Parental type 2 diabetes has been associated with older age at diagnosis of type 1 diabetes in the offspring [8] but there are scarce data about the influence of family history of type 2 diabetes in the disease process before clinical diagnosis of type 1 diabetes.

The Environmental Determinants of Diabetes in the Young (TEDDY) study is an international prospective cohort study that aims to identify environmental factors that trigger or protect against the development of islet autoimmunity and type 1 diabetes. We analysed the TEDDY cohort to determine whether family history of type 1 diabetes, type 2 diabetes, or any autoimmune disease in FDRs and SDRs was associated with the development of islet autoimmunity or progression to type 1 diabetes in young children.

## Methods

In 2004–2010, the TEDDY study screened 424,788 newborns for *HLA-DR-DQ* genotypes conferring increased risk for type 1 diabetes. Sex was assigned at birth and both boys and girls were equally invited to the study. A total of 8676 children were enrolled and invited to follow-up for development of islet autoimmunity and type 1 diabetes in clinical centres in Finland, Germany, Sweden and the USA [9, 10]. This analysis was based on the TEDDY database cut-off of 31 January 2016. The information about FDRs with type 1 diabetes was obtained from either the Infant Screening Form or Family History Questionnaires and was available for 8558 children. The information about type 1 diabetes in SDRs, and data for other diabetes types and other autoimmune diseases (see electronic supplementary material [ESM] Methods) in FDRs and SDRs was obtained from Family History Questionnaires, available from 7479 children. In the full cohort, 2454 out of 7479 children had either an FDR or an SDR with type 2 diabetes. This included 34 children with an FDR but not SDR with type 2 diabetes. There were 669 children with persistent confirmed islet autoimmunity, but for three of these children information on type 2 diabetes in SDRs was not available. Among the remaining children with persistent confirmed islet autoimmunity, 230 out of 666 children had either an FDR or an SDR with type 2 diabetes. This included five children with an FDR but not an SDR with type 2 diabetes, thus leaving 225 children with, and 441 without, an SDR with type 2 diabetes. The median follow-up time of these children after seroconversion was 6.5 years (IQR 3.3–8.5). The characteristics of TEDDY participants with or without an SDR with type 2 diabetes are described in ESM Table 1, and the characteristics of those who had persistent confirmed islet autoimmunity are shown in ESM Table 2.

Written informed consent was obtained for all study participants from the parents or legal guardians, and the children at an appropriate age gave their assent. The study was approved by local ethics committees or institutional review boards.

### HLA genotyping

The children from the general population (GP) were screened for the following high-risk genotypes: *DR3/4*; *DR4/4*; *DR4/8* and *DR3/3* [11, 12]. Children having an FDR with type 1 diabetes were additionally screened for the following five genotypes: *DR4/4b*; *DR4/1*; *DR4/13*; *DR4/9*; and *DR3/9* [11, 12].

### Data collection

The Infant Screening Form was used when the child was aged 3 months to collect information about the presence of type 1 diabetes in FDRs. The Family History Questionnaire was filled out when the child was aged 9 months and every 4 years thereafter. This questionnaire collected information about the presence of any type of diabetes (type 1 diabetes, type 2 diabetes, MODY, gestational diabetes, unknown diabetes type) and autoimmune diseases (see ESM Methods) among the FDRs (mother, father, full sibling) and SDRs (grandparents, half siblings, maternal and paternal siblings). In addition, TEDDY families were asked at yearly intervals whether they had done anything to prevent diabetes (yes or no).

### Islet autoantibodies

IAA, GADA and IA-2A were measured by radio-binding assays in serum samples collected every 3 months for the first 4 years and biannually thereafter unless the child developed autoantibodies, after which the collection was continued at intervals of 3 months. Autoantibodies were measured at the University of Colorado, Denver and at the University of Bristol, UK [13]. Positive samples were reanalysed at the second reference laboratory for confirmation. The positivity for islet autoimmunity (persistent confirmed islet autoantibodies) was defined as a positive result at both reference laboratories and by the presence of at least one islet autoantibody (GADA, IA-2A or IAA) on at least two consecutive visits. The date of seroconversion to islet autoimmunity was defined as the date of the first of the two consecutive positive samples. Children with islet autoantibodies possibly derived from maternal IgG transmission were not considered autoantibody positive unless the child tested negative before his/her own positive sample, or the autoantibody persisted beyond 18 months of age [13].

### Statistical methods

Specific variables were created to indicate whether the FDRs and SDRs had type 1 diabetes, type 2 diabetes, gestational diabetes, other type of diabetes, or any of the following autoimmune diseases: coeliac disease; inflammatory bowel disease; lupus; psoriasis; rheumatoid arthritis; or thyroid autoimmune diseases (hypo- or hyperthyroidism). All other autoimmune diseases were pooled in an ‘other autoimmune disease’ category. Because of the sparsity of the autoimmune diseases, these were pooled over both FDRs and SDRs, resulting in a variable indicating any family history of autoimmune disease other than type 1 diabetes. The regression analysis was first conducted by using the variables indicating family history of diabetes. If any of these variables was found to be associated with the endpoint, it was included in subsequent analyses of autoimmune disease variables.

#### Islet autoimmunity

The role of family history of diabetes and autoimmune diseases was analysed via proportional hazards regression when the endpoint was the development of persistent confirmed islet autoantibodies. Time was defined as the age of the participant. Previous factors found to be associated with development of islet autoantibodies were class II HLA, sex, FDR with type 1 diabetes, SNPs (rs2476601, rs2816316, rs2292239, rs3184504, rs4948088, rs1004446, rs1270876 and rs10517086) and probiotic use prior to 3 months of age (yes or no) [14]. For each SNP, the number of minor alleles was the covariate. These variables were automatically included in the proportional hazards regression analysis stratified by country. The set of variables indicating family history of diabetes and autoimmune diseases was examined in a backward selection approach with a *p* value cut-off of 0.01.

#### Progression to type 1 diabetes

In participants who developed persistent confirmed islet autoantibodies, the role of family history of diabetes and autoimmune diseases was analysed via proportional hazards regression where the endpoint was the diagnosis of type 1 diabetes. The time was defined as the time from development of autoantibodies until the diagnosis. Previous factors found to be associated with progression to type 1 diabetes were class II HLA, age at seroconversion, FDR with type 1 diabetes, SNPs (rs2476601, rs2292239, rs3184504, rs1004446, rs7111341, rs11711054 and rs3825932) and mean IAA and IA-2A levels during exposure time [15, 16]. For each SNP, the number of minor alleles was the covariate. These variables were automatically included in the proportional hazards regression analysis stratified by country. The set of variables indicating family history of diabetes and autoimmune diseases were examined in a backward selection approach with a *p* value cut-off of 0.01.

## Results

The frequencies of TEDDY children having either an FDR or an SDR with diabetes (type 1 diabetes, type 2 diabetes, gestational diabetes, unknown type of diabetes) or autoimmune diseases are shown in Table 1. Family history of type 1 diabetes was positive in 11.3% and 9.9% of TEDDY children for their FDRs and SDRs, respectively. Family history of type 2 diabetes in FDRs was positive for only 1.7% of TEDDY children while 32.4% of them had at least one SDR with type 2 diabetes. Gestational diabetes had been diagnosed in 6.8% of the mothers of all TEDDY children.

**Table 1:** Proportion of TEDDY children with a family history of various types of diabetes and autoimmune disorders in their FDRs and SDRs

Country	USA	Finland	Germany	Sweden	Total^a^
No. of children^a^	3059	1649	485	2286	7479
Type 1 diabetes, *N* (%)					
FDR^b^	393 (10.7)	181 (10.0)	215 (37.0)	174 (6.9)	963 (11.3)
SDR	278 (9.1)	175 (10.6)	53 (10.9)	232 (10.1)	738 (9.9)
Type 2 diabetes, *N* (%)					
FDR	70 (2.3)	22 (1.3)	4 (0.8)	28 (1.2)	124 (1.7)
SDR	1093 (35.7)	559 (33.9)	143 (29.5)	625 (27.3)	2420 (32.4)
Gestational diabetes, *N* (%)					
FDR	187 (6.1)	224 (13.6)	21 (4.3)	74 (3.2)	506 (6.8)
SDR	146 (4.8)	86 (5.2)	14 (2.9)	60 (2.6)	306 (4.1)
Diabetes, unknown type, *N* (%)					
FDR	42 (1.4)	7 (0.4)	3 (0.6)	4 (0.2)	56 (0.7)
SDR	255 (8.3)	47 (2.9)	26 (5.4)	144 (6.3)	472 (6.3)
Thyroid disease, *N* (%)					
FDR	280 (9.2)	108 (6.5)	75 (15.5)	149 (6.5)	612 (8.2)
SDR	552 (18.0)	380 (23.0)	110 (22.7)	410 (17.9)	1452 (19.4)
Rheumatoid arthritis, *N* (%)					
FDR	44 (1.4)	42 (2.5)	5 (1.0)	52 (2.3)	143 (1.9)
SDR	319 (10.4)	291 (17.6)	44 (9.1)	297 (13.0)	951 (12.7)
Psoriasis, *N* (%)					
FDR	109 (3.6)	72 (4.4)	23 (4.7)	120 (5.2)	324 (4.3)
SDR	187 (6.1)	233 (14.1)	46 (9.5)	403 (17.6)	869 (11.6)
Lupus, *N* (%)					
FDR	14 (0.5)	4 (0.2)	0 (0.0)	2 (0.1)	20 (0.3)
SDR	98 (3.2)	5 (0.3)	2 (0.4)	11 (0.5)	116 (1.6)
Coeliac disease, *N* (%)					
FDR	59 (1.9)	85 (5.2)	5 (1.0)	93 (4.1)	242 (3.2)
SDR	85 (2.8)	217 (13.2)	5 (1.0)	107 (4.7)	414 (5.5)
Inflammatory bowel disease, *N* (%)					
FDR	35 (1.1)	37 (2.2)	6 (1.2)	51 (2.2)	129 (1.7)
SDR	131 (4.3)	99 (6.0)	9 (1.9)	112 (4.9)	351 (4.7)
Other autoimmune disease, *N* (%)					
FDR	118 (3.9)	105 (6.4)	38 (7.8)	108 (4.7)	369 (4.9)
SDR	281 (9.2)	283 (17.2)	70 (14.4)	315 (13.8)	949 (12.7)

Information on type 1 diabetes in FDRs and SDRs, and information on all other diseases in both FDRs and SDRs, is based on Family History Questionnaires that were available from 7479 children. SDRs include grandparents, aunts and uncles of the TEDDY child

^a^No. of children with information about both FDR and SDR

^b^Information about FDRs (mother, father or siblings of the TEDDY child) was obtained from either the Infant Screening Form or Family History Questionnaires and was available for 3665 children in the USA, 1808 in Finland, 581 in Germany and 2504 in Sweden (a total of 8558 children)

### Effect of family history on islet autoimmunity

The positive FDR status for type 1 diabetes significantly increased the risk of the child for developing islet autoantibodies as compared with children with no FDR with type 1 diabetes (HR 2.2 [95% CI 1.8, 2.8]; *p*<0.001). A secondary analysis was run partitioning the FDR category into mother, father and/or sibling. The risks associated with a father (HR 2.5 [95% CI 1.9, 3.2]; *p*<0.001) and a sibling with type 1 diabetes (HR 3.5 [95% CI 2.5, 4.8]; *p*<0.001) were higher than for a mother with the disease (HR 1.4 [95% CI 0.9, 2.0]; *p*=0.106). Family history of type 2 diabetes, gestational diabetes, other type of diabetes or autoimmune diseases in FDRs or SDRs was not associated with the development of islet autoantibodies.

Because of the strong influence of the positive FDR status for type 1 diabetes, we also examined the subgroup of participants who did not have an FDR with type 1 diabetes, referred to as GP children (*N*=6516). In this analysis, children having an SDR with type 1 diabetes had an HR of 1.4 (95% CI 1.1, 1.8; *p*=0.017) for the development of islet autoimmunity. No other type of diabetes or autoimmune disease in the family history was associated with the development of islet autoimmunity in the GP subgroup.

### Effect of family history on progression from islet autoimmunity to clinical type 1 diabetes

**Fig. 1:**
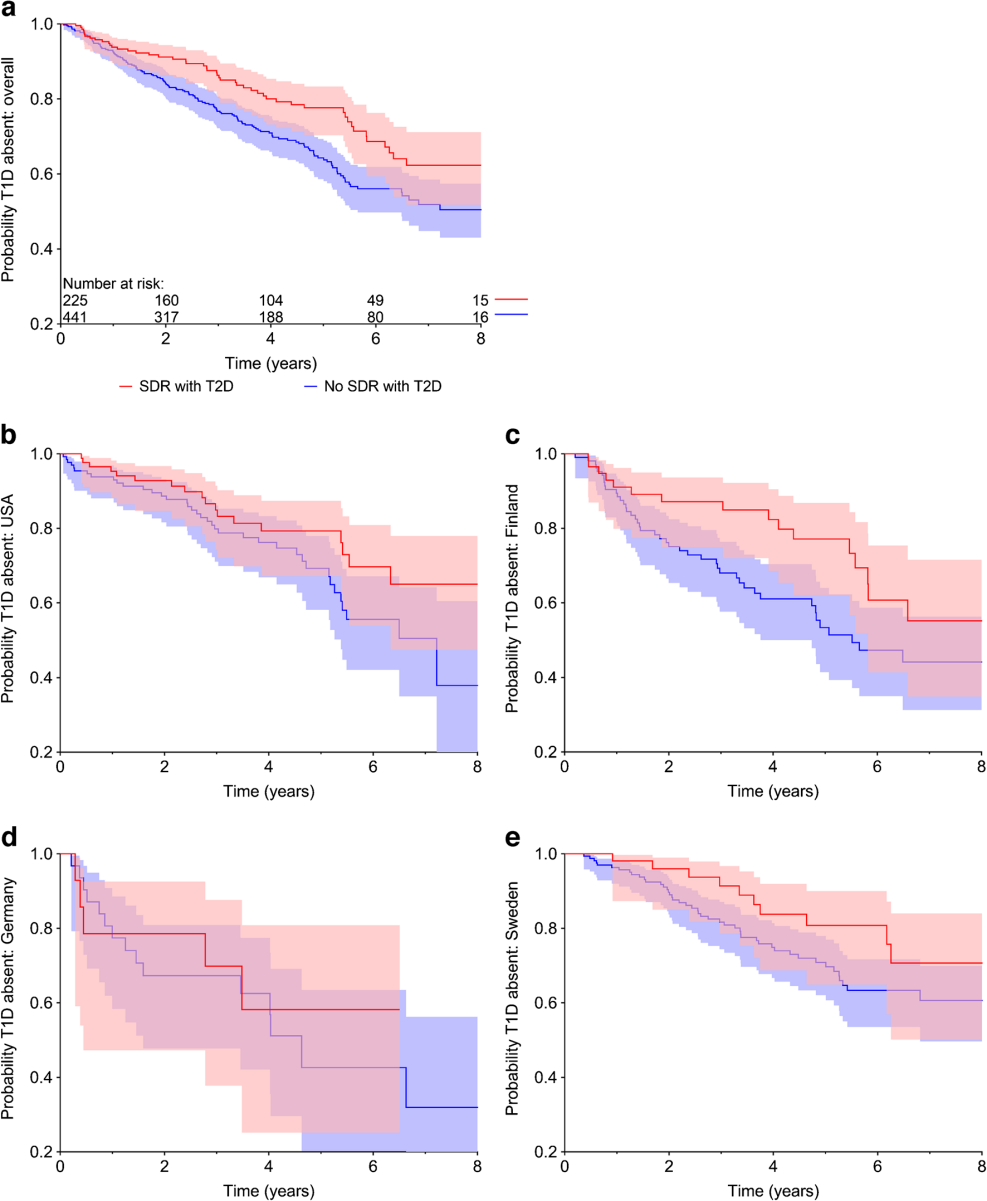
Probability of remaining free of type 1 diabetes in young children with persistent confirmed islet autoimmunity. Participants with and without an SDR with type 2 diabetes are compared (red and blue, respectively; *p*=0.004). (**a**) All children with persistent confirmed islet autoimmunity are included (*n*=666, median follow-up time since seroconversion 6.5 years). (**b**–**e**) Country-specific comparisons are shown. Shaded areas indicate 95% CIs for the cumulative probabilities. T1D, type 1 diabetes; T2D, type 2 diabetes

Persistent islet autoantibodies were observed in 669 children (Table 2). Of these, 616 (92%) had complete covariate information including family history of diabetes and autoimmune diseases. At the time of the data cut-off, a total of 233 TEDDY participants had been diagnosed with type 1 diabetes. Analysis of the influence of family history of diabetes or autoimmune disease on progression to clinical disease revealed that type 2 diabetes in SDRs significantly delayed progression from islet autoimmunity to type 1 diabetes (HR 0.61 [95% CI 0.44, 0.86]; *p*=0.004) (Fig. 1). For example, the time point at which 80% of children with persistent islet autoimmunity remained free of type 1 diabetes occurred 14.3 months later in participants having SDR with type 2 diabetes compared with those who did not have SDR with type 2 diabetes. The variables for ‘FDR with type 2 diabetes’ and ‘SDR with type 2 diabetes’ were pooled and a single combined variable for ‘FDR or SDR with type 2 diabetes’ was created. In the proportional hazards model the combined variable was statistically significantly associated with decreased risk of progression from islet autoimmunity to clinical type 1 diabetes (HR 0.62; *p*=0.005).

**Table 2:** The effect of type 2 diabetes in SDRs on progression to type 1 diabetes in TEDDY children with various first autoantibody presentation as analysed by proportional hazards regression

Autoantibody profile	*N*^a^	HR (95% CI)	*p* value
IAA only	264	0.53 (0.31, 0.92)	0.023
GADA only	275	0.49 (0.23, 1.05)	0.068
IAA and GADA together	86	0.75 (0.29, 1.95)	0.556
IAA only + IAA and GADA	350	0.61 (0.40, 0.93)	0.022
GADA only + IAA and GADA	361	0.87 (0.53, 1.44)	0.593
All persistent confirmed autoantibodies^b^	669	0.61 (0.44, 0.86)	0.004

HRs with 95% CIs are shown for islet autoantibody positive children who had SDRs with type 2 diabetes vs those without such relatives

^a^No. of children

^b^Also includes children with IA-2A as the first or one of the first autoantibodies

To further study the effect of type 2 diabetes in SDRs on progression, we analysed separate subgroups of children with different first-appearing autoantibodies or their combinations and found that the effect was significant in the subgroup with IAA only as the first autoantibody but the magnitude of the HR was similar in the GADA-only subgroup albeit not statistically significant (Table 2). When the subgroups with IAA only and IAA together with GADA were combined, the effect was also significant.

The 5-year cumulative probability of type 1 diabetes after seroconversion was 24% in children who had SDR with type 2 diabetes compared with 38% in children without such family history (Table 3). The reduction in the probability of type 1 diabetes was consistent in all TEDDY countries, with the largest difference between the groups in Finland (Table 3, Fig. 1c). If all SDRs with an unknown diabetes type were assumed to have type 2 diabetes, the HR slightly increased to 0.7 (95% CI 0.5, 0.9; *p*=0.016). No other variable indicating family history of diabetes or autoimmune disease was associated with progression to clinical disease.

**Table 3:** Cumulative probability (%) of type 1 diabetes 5 years after seroconversion for persistent confirmed islet autoantibodies estimated by Kaplan–Meier analysis

Type 2 diabetes in SDR	USA	Finland	Germany	Sweden	Total
Not present, % (SE %)^a^	32 (5)	47 (6)	60 (11)	30 (4)	38 (3)
Present, % (SE %)	24 (5)	23 (6)	42 (15)	21 (6)	24 (3)

^a^SEs in parentheses are from Greenwood estimate

To study the putative mechanisms that might explain the delaying effect of type 2 diabetes in SDRs on progression, we analysed whether the effect was different between the families who tried actively to do something to prevent diabetes compared with those who did not. Across all countries, 78% of families indicated they had done something to prevent diabetes at some time point and 69% indicated they had done something to prevent the disease after they had learned about their child’s autoantibodies. Neither of these actions affected progression and when either of these variables was included in the Cox regression model, the delaying effect of type 2 diabetes in SDRs remained significant (*p*<0.01).

The effect of diabetes and autoimmune disease family history on progression from islet autoimmunity to type 1 diabetes was also analysed separately in FDRs and GP children. No additional family history factor had a statistically significant effect in either of the subgroups. Although type 2 diabetes in SDRs was not statistically significant in either subgroup alone, the trend and magnitude of the effect was consistent across both subgroups, explaining the significance in the total cohort of progressors.

## Discussion

Our results indicate that the family history of type 1 and type 2 diabetes associate differently with development of islet autoimmunity and progression to type 1 diabetes, the two phases of the disease process. Most intriguingly, type 2 diabetes in SDRs of TEDDY children was associated with slower progression from autoimmunity to type 1 diabetes as compared with those without such family history. Consistency of this finding in all four TEDDY countries further supported the validity of this association. It is also in line with earlier studies reporting that parental type 2 diabetes is associated with older age at diagnosis of type 1 diabetes in the offspring [8, 17–20]. Our analyses also confirmed an increased risk of islet autoimmunity in children who have an FDR with type 1 diabetes [13, 14]. More specifically, those with a father or a sibling with type 1 diabetes had a higher risk for islet autoimmunity than children having a mother with type 1 diabetes. Furthermore, in participants without an affected FDR, the presence of type 1 diabetes in SDRs was also associated with an increased risk for islet autoimmunity.

Most previous family history studies on the development of type 1 diabetes have been cross-sectional case–control series and have not distinguished between the development of islet autoimmunity and progression to clinical diabetes. In addition, retrospective studies suffer from more severe recall bias than prospective studies. The TEDDY study, with its intensive follow-up design and prospective data collection including extended family disease history of both FDRs and SDRs, provided us with a unique opportunity to investigate both islet autoimmunity and progression to clinical diagnosis. Earlier studies have shown that different genetic variants are associated with differential rates of developing islet autoimmunity as well as progression to type 1 diabetes [15, 16, 21–23]. Our data expand on those observations to imply that family-history-associated risk factors are also distinct for the two disease phases.

Extensive case–control studies have revealed that susceptibility genes for type 1 diabetes and type 2 diabetes are different. Most of the genes associated with type 1 diabetes are involved with immune function [24], whereas most type-2-diabetes-associated genes affect beta cell function or insulin sensitivity [25]. Our new finding that type 2 diabetes in SDRs is associated with delayed progression points to the possibility that type 2 diabetes risk genes could influence the progression phase of type 1 diabetes. It is noteworthy that in the early phases of type 2 diabetes, there is hyperinsulinaemia preceded by insulin resistance while beta cell failure is a late phenomenon. Reduced insulin clearance might also contribute to protection against progression to clinical type 1 diabetes. We hypothesise that children having a family member with type 2 diabetes might have enhanced beta cell functional capacity that is linked to their genetic background. This raises the question of whether the genes associated with type 2 diabetes maintain or promote beta cell function in individuals with islet autoimmunity. Previous analysis of 12 SNPs in selected type 2 diabetes risk loci found no associations between the investigated SNPs and the development of islet autoimmunity or type 1 diabetes, perhaps due to insufficient sample size [26]. No family history data on type 2 diabetes was included [26]. Even the current TEDDY dataset may be too small to give an unambiguous answer to the question of whether any of the more than 100 genetic risk variants for type 2 diabetes would influence the progression rate to type 1 diabetes, and whether the delay in progression to type 1 diabetes associated with family history of type 2 diabetes would be explained by type 2 diabetes risk genes. Large datasets, perhaps combined from several prospective studies that follow participants from islet autoimmunity until diagnosis of type 1 diabetes and have data on family history of type 2 diabetes, are required to clarify these questions.

Which other factors could explain the association between family history of type 2 diabetes and delayed progression? The present data refuted the possibility that dietary or lifestyle changes introduced by the families having relatives with type 2 diabetes would offer explanation.

Heterogeneity of the disease process of type 1 diabetes has been recognised in prospective cohort studies characterising two main pathways in the initiation of islet autoimmunity: (1) IAA as the first-appearing islet autoantibody associated with very young age and the *HLA-DR3/4*, *HLA-DR4/4* and *HLA-DR4/8* genotypes; or (2) GADA as the first autoantibody associated with older age at presentation and the *HLA-DR3/3* genotype [13, 14, 27]. The subgroup analysis taking into account the first-appearing autoantibody showed that in both endotypes the presence of type 2 diabetes in SDRs delayed the progression phase rather similarly (Table 2).

In addition to the genetic factors, previous studies have found that age at multiple autoantibody positivity, type of the first-appearing autoantibody, levels of autoantibodies, and sex were significantly associated with progression to clinical type 1 diabetes [15, 21, 23, 28, 29]. Here, we show for the first time that type 2 diabetes in SDRs was inversely correlated to the progression rate from islet autoimmunity to type 1 diabetes. The association is probably not specific for SDRs only but most probably applies to FDR too. This was supported by a similar result in an analysis where family history of type 2 diabetes in FDRs or SDRs was combined. However, only 1.7% of TEDDY children had an FDR with type 2 diabetes, whereas 32.4% had an SDR with type 2 diabetes, which allowed meaningful statistical comparison only for the combined group or the SDR group alone. All in all, our finding suggests that extended family history is highly valuable in the search for risk determinants of type 1 diabetes.

In contrast to the association between type 2 diabetes in SDRs and progression rate, the family history of type 2 diabetes was not associated with development of islet autoimmunity. We confirmed the previous observation that type 1 diabetes in FDRs, especially in a father or a sibling, increases the risk of islet autoimmunity [14]. Here, we showed that type 1 diabetes in SDRs also significantly increased the risk of autoimmunity in GP children, in line with the results from a Finnish case–control study [2].

The limitations of our study include the fact that the study population represents children with selected *HLA-DR-DQ* genotypes and countries of origin, which limits generalisability of the results. Data on the family history of autoimmune diseases were collected at study visits by using questionnaires, a method which may have caused inaccuracies due to the rarity of these diseases and lack of familiarity with them among the parents of TEDDY children. Extension of family history data collection to SDRs may increase uncertainty of diabetes types, such as latent autoimmune diabetes in adults (LADA) [30]. However, the number of FDRs and SDRs possibly having LADA should be very small, since about 3–12% of all diabetes in adults can be classified as LADA [31]. While large registry-based studies have shown modestly significant associations between type 1 diabetes and several autoimmune diseases other than type 1 diabetes in the family, we could not observe such associations, suggesting that even large cohort studies cover too small a population to demonstrate a robust association with rare diseases [32].

In conclusion, diabetes-related family history is differentially associated with the two phases of type 1 diabetes pathogenesis. Type 1 diabetes in the family history is associated with an increased risk of developing islet autoimmunity whereas type 2 diabetes is associated with a slower rate of progression from islet autoimmunity to clinical diagnosis. These data can be used to generate new hypotheses to dissect the genetic and environmental determinants of type 1 diabetes pathogenesis.

## Supplementary Information

Below is the link to the electronic supplementary material.

ESM (PDF 422 KB)

## Data Availability

Data reported here can be obtained by request at the NIDDK Central Repository website, Resources for Research (R4R), https://repository.niddk.nih.gov/.

## Abbreviations

FDR: First-degree relative
GP: General population
LADA: Latent autoimmune diabetes in adults
SDR: Second-degree relative
TEDDY: The Environmental Determinants of Diabetes in the Young
