# Supplementary material for: Family history of type 2 diabetes delays development of type 1 diabetes in TEDDY children with islet autoimmunity
Source: Diabetologia. 2025 Dec 8;69(3):643–52. doi: 10.1007/s00125-025-06613-1 (PMC12881113; doi:10.1007/s00125-025-06613-1)
Supplement: Supplementary file 1 — ESM (PDF 422 KB) [file 125_2025_6613_MOESM1_ESM.pdf]

Electronic Supplementary Materials

Riitta Veijola, Roy N. Tamura, Joanna L. Clasen, Helena Elding Larsson, Katharina Warncke, Andrea K. Steck, Michael J. Haller, Berglind Jonsdottir, Beena Akolkar, William A. Hagopian, Marian J. Rewers, Jin-Xiong She, Anette-Gabriele Ziegler, Jeffrey P. Krischer, and Jorma Toppari, TEDDY Study Group

Family history of type 2 diabetes delays development of type 1 diabetes in TEDDY children with islet autoimmunity

ESM Methods

Autoimmune diseases in the family……………………………………………………. Page 2

ESM Table 1 ……………………………………………………………………………………………… Page 3

ESM Table 2 ……………………………………………………………………………………………… Page 5

TEDDY Study Group ……………………………………………………………………………….. Page 7

Abbreviations

BMI	Body mass index
IA	Islet autoimmunity
T1D	Type 1 diabetes
T2D	Type 2 diabetes

ESM Methods

TEDDY families were asked to fill out a questionnaire that collected information about the presence of the following autoimmune diseases in the family, among the FDRs and SDRs.

1) Graves disease (Hyperthyroid-High)
2) Hyperthyroid, not confirmed Graves
3) Hashimoto's disease (Hypothyroid-Low)
4) Rheumatoid arthritis
5) Juvenile rheumatoid arthritis
6) Coeliac disease
7) Myasthenia gravis
8) Addison's disease
9) Pernicious anaemia
10) Lupus
11) Ulcerative colitis
12) Crohn's disease
13) Multiple sclerosis
14) Ankylosing spondylitis
15) Juvenile ankylosing spondylitis
16) Psoriasis
17) Vitiligo
18) Wegener's granulomatosis or syndrome
19) Scleroderma
20) Alopecia areata (patchy hair loss)
21) Alopecia totalis (entire scalp hair loss)
22) Alopecia universalis (loss of all body hair)
23) Sjögren's syndrome
24) Other, specify

ESM Table 1

Characteristics of the TEDDY participants with or without a second-degree relative (SDR) with type 2 diabetes (T2D)

**Characteristic**	**No SDR with T2D (N=5059)**	**SDR with T2D (N=2420)**	**Total (N=7479)**	***P* value**
IA persistent positive				0.410
Yes	441 (8.7%)	225 (9.3%)	666 (8.9%)	
No	4618 (91.3%)	2195 (90.7%)	6813 (91.1%)	
Country				< 0.001
USA	1966 (38.9%)	1093 (45.2%)	3059 (40.9%)	
Finland	1090 (21.5%)	559 (23.1%)	1649 (22.0%)	
Germany	342 (6.8%)	143 (5.9%)	485 (6.5%)	
Sweden	1661 (32.8%)	625 (25.8%)	2286 (30.6%)	
Sex				0.573
Female	2465 (48.7%)	1196 (49.4%)	3661 (49.0%)	
Male	2594 (51.3%)	1224 (50.6%)	3818 (51.0%)	
HLA				0.674
DR3/4	1965 (38.8%)	953 (39.4%)	2918 (39.0%)	
DR4/4	987 (19.5%)	483 (20.0%)	1470 (19.7%)	
FDR-specific	176 (3.5%)	75 (3.1%)	251 (3.4%)	
DR4/8	867 (17.1%)	428 (17.7%)	1295 (17.3%)	
DR3/3	1064 (21.0%)	481 (19.9%)	1545 (20.7%)	
Probiotic use prior to 3 months of age				0.115
No	3310 (65.4%)	1628 (67.3%)	4938 (66.0%)	
Yes	1749 (34.6%)	792 (32.7%)	2541 (34.0%)	
rs1004446_a[1]				0.171
0	1794 (39.6%)	889 (40.7%)	2683 (39.9%)	
1	2091 (46.1%)	1017 (46.6%)	3108 (46.3%)	
2	651 (14.4%)	277 (12.7%)	928 (13.8%)	
rs10517086_a				0.427
0	2291 (50.5%)	1135 (52.0%)	3426 (51.0%)	
1	1874 (41.3%)	884 (40.5%)	2758 (41.0%)	
2	371 (8.2%)	164 (7.5%)	535 (8.0%)	
rs12708716_g				0.703
0	2020 (44.5%)	951 (43.6%)	2971 (44.2%)	
1	1990 (43.9%)	981 (44.9%)	2971 (44.2%)	
2	526 (11.6%)	251 (11.5%)	777 (11.6%)	

Characteristic	No SDR with T2D (N=5059)	SDR with T2D (N=2420)	Total (N=7479)	*P* value
rs2292239_a				0.078
0	2023 (44.6%)	1036 (47.5%)	3059 (45.5%)	
1	2051 (45.2%)	928 (42.5%)	2979 (44.3%)	
2	462 (10.2%)	219 (10.0%)	681 (10.1%)	
rs2476601_a				0.821
0	3595 (79.3%)	1734 (79.4%)	5329 (79.3%)	
1	884 (19.5%)	418 (19.1%)	1302 (19.4%)	
2	57 (1.3%)	31 (1.4%)	88 (1.3%)	
rs2816316_c				0.932
0	3041 (67.0%)	1458 (66.8%)	4499 (67.0%)	
1	1338 (29.5%)	652 (29.9%)	1990 (29.6%)	
2	157 (3.5%)	73 (3.3%)	230 (3.4%)	
rs3184504_a				0.961
0	1397 (30.8%)	679 (31.1%)	2076 (30.9%)	
1	2230 (49.2%)	1071 (49.1%)	3301 (49.1%)	
2	909 (20.0%)	433 (19.8%)	1342 (20.0%)	
rs4948088_a				0.657
0	4124 (90.9%)	1999 (91.6%)	6123 (91.1%)	
1	398 (8.8%)	177 (8.1%)	575 (8.6%)	
2	14 (0.3%)	7 (0.3%)	21 (0.3%)	

[1]N = 6719 children not missing SNP data

ESM Table 2

Characteristics of the TEDDY participants who had persistent confirmed islet autoimmunity (IA). Children with or without second-degree relative (SDR) with type 2 diabetes (T2D) shown separately.

Characteristic	No SDR with T2D (N=441)	SDR with T2D (N=225)	Total (N=666)	*P* value
T1D				0.008
No	289 (65.5%)	170 (75.6%)	459 (68.9%)	
Yes	152 (34.5%)	55 (24.4%)	207 (31.1%)	
First appearing islet autoantibody				0.593
IA-2A only	11 (2.5%)	4 (1.8%)	15 (2.3%)	
GADA only	179 (40.6%)	96 (42.7%)	275 (41.3%)	
GADA and IA-2A	3 (0.7%)	2 (0.9%)	5 (0.8%)	
IAA only	173 (39.2%)	88 (39.1%)	261 (39.2%)	
IAA and IA-2A	7 (1.6%)	0 (0.0%)	7 (1.1%)	
IAA and GADA	58 (13.2%)	28 (12.4%)	86 (12.9%)	
IAA, GADA and IA-2A	10 (2.3%)	7 (3.1%)	17 (2.6%)	
IA seroconversion age (years)				0.188
Median	2.4	2.8	2.5	
IQR	3.2	3.7	3.3	
Time from IA seroconversion to T1D diagnosis (years)				0.727
Median	2.2	2.8	2.4	
IQR	3.0	3.5	3.1	
T1D diagnosis age (years)				0.452
Median	4.3	4.6	4.3	
IQR	3.5	4.8	3.6	
Country				0.003
USA	134 (30.4%)	93 (41.3%)	227 (34.1%)	
Finland	106 (24.0%)	62 (27.6%)	168 (25.2%)	
Germany	33 (7.5%)	14 (6.2%)	47 (7.1%)	
Sweden	168 (38.1%)	56 (24.9%)	224 (33.6%)	
Sex				0.440
Female	204 (46.3%)	97 (43.1%)	301 (45.2%)	
Male	237 (53.7%)	128 (56.9%)	365 (54.8%)	
HLA				0.607
DR3/4	213 (48.3%)	119 (52.9%)	332 (49.8%)	
DR4/4	83 (18.8%)	37 (16.4%)	120 (18.0%)	
FDR-specific	19 (4.3%)	6 (2.7%)	25 (3.8%)	
DR4/8	75 (17.0%)	34 (15.1%)	109 (16.4%)	
DR3/3	51 (11.6%)	29 (12.9%)	80 (12.0%)	

Characteristic	No SDR with T2D (N=441)	SDR with T2D (N=225)	Total (N=666)	*P* value
BMI at IA seroconversion				0.370
Median	16.6	16.5	16.5	
IQR	1.9	2.1	2.0	
Coeliac disease				0.481
No	414 (93.9%)	208 (92.4%)	622 (93.4%)	
Yes	27 (6.1%)	17 (7.6%)	44 (6.6%)	
rs1004446_a[1]				0.291
0	186 (45.0%)	88 (43.1%)	274 (44.4%)	
1	183 (44.3%)	101 (49.5%)	284 (46.0%)	
2	44 (10.7%)	15 (7.4%)	59 (9.6%)	
rs2292239_a				0.990
0	160 (38.7%)	78 (38.2%)	238 (38.6%)	
1	202 (48.9%)	101 (49.5%)	303 (49.1%)	
2	51 (12.3%)	25 (12.3%)	76 (12.3%)	
rs2476601_a				0.963
0	288 (69.7%)	143 (70.1%)	431 (69.9%)	
1	118 (28.6%)	57 (27.9%)	175 (28.4%)	
2	7 (1.7%)	4 (2.0%)	11 (1.8%)	
rs3184504_a				0.486
0	95 (23.0%)	50 (24.5%)	145 (23.5%)	
1	209 (50.6%)	93 (45.6%)	302 (48.9%)	
2	109 (26.4%)	61 (29.9%)	170 (27.6%)	
rs7111341_a				0.608
0	235 (56.9%)	114 (55.9%)	349 (56.6%)	
1	156 (37.8%)	75 (36.8%)	231 (37.4%)	
2	22 (5.3%)	15 (7.4%)	37 (6.0%)	
rs11711054_g				0.806
0	197 (47.7%)	99 (48.5%)	296 (48.0%)	
1	177 (42.9%)	89 (43.6%)	266 (43.1%)	
2	39 (9.4%)	16 (7.8%)	55 (8.9%)	
rs3825932_a				0.146
0	197 (47.7%)	81 (39.7%)	278 (45.1%)	
1	161 (39.0%)	95 (46.6%)	256 (41.5%)	
2	55 (13.3%)	28 (13.7%)	83 (13.5%)	

[1]N = 616 children not missing SNP data

The TEDDY Study Group

Colorado Clinical Center: Marian Rewers, M.D., Ph.D., PI[1,4,6,9,10], Kimberly Bautista[11], Judith Baxter[8,911], Daniel Felipe-Morales, Brigitte I. Frohnert, M.D., Ph.D.[2,13], Marisa Stahl, M.D.[12], Patricia Gesualdo[2,6,11,13], Michelle Hoffman[11,12,13], Randi Johnson, Ph.D.[2,3], Rachel Karban[11], Edwin Liu, M.D.[12], Jill Norris, Ph.D.[2,3,11], Holly O'Donnell, Ph.D.[8], Andrea Steck, M.D.[3,13], Kathleen Waugh[6,7,11].

University of Colorado, Anschutz Medical Campus, Barbara Davis Center for Childhood Diabetes, Aurora, CO, USA.

Finland Clinical Center: Jorma Toppari, M.D., Ph.D., PI[¥^1,4,10,13], Olli G. Simell, M.D., Ph.D., Annika Adamsson, Ph.D.[^11], Suvi Ahonen*[±§], Mari Åkerlund*[±§], Sirpa Anttila[μ¤], Leena Hakola, Ph.D.*[±], Sanni Heikura[μ¤], Tiia Honkanen[μ¤], Heikki Hyöty, M.D., Ph.D.*[±6], Jorma Ilonen, M.D., Ph.D.[¥3], Saori Itoshima, M.D.[¥^], Sanna Jokipuu[^], Taru Karjalainen[μ¤], Leena Karlsson[^], Jukka Kero, M.D., Ph.D.[¥^3, 13], Marika Korpela[μ¤], Jaakko J. Koskenniemi M.D., Ph.D.[¥^], Miia Kähönen[μ¤11,13], Mikael Knip, M.D., Ph.D.*[±], Minna-Liisa Koivikko[μ¤], Katja Kokkonen*[±], Merja Koskinen*[±], Mirva Koreasalo*[±§2], Kalle Kurppa, M.D., Ph.D.*[±12], Salla Kuusela, M.D. [μ¤], Jutta Laiho, Ph.D.*[6], Tiina Latva-aho[μ¤], Laura Leppänen[^], Katri Lindfors, Ph.D.*[12], Maria Lönnrot, M.D., Ph.D.*[±6], Elina Mäntymäki[^], Markus Mattila, Ph.D.*[±2], Maija E. Miettinen, Ph.D.[§2], Teija Mykkänen[μ¤], Tiina Niininen[±]*[11], Sari Niinistö, Ph.D.[§2], Noora Nurminen[*±], Sami Oikarinen, Ph.D.*[±6], Hanna-Leena Oinas*[±], Paula Ollikainen[μ¤], Zhian Othmani[¥], Sirpa Pohjola [μ¤], Jenna Rautanen[§], Mia Rein[μ¤], Minna Romo[^], Juulia Rönkä[μ¤], Nelli Rönkä[μ¤], Noora Ruotsalainen[μ¤], Satu Simell, M.D., Ph.D.[¥12], Päivi Tossavainen, M.D.[μ¤], Mari Vähä-Mäkilä[¥], Eeva Varjonen[^11], Riitta Veijola, M.D., Ph.D.[μ¤13], Irene Viinikangas[μ¤], Suvi M. Virtanen, M.D., Ph.D.*[±§2].

[¥]University of Turku, Turku, Finland, *Tampere University, Tampere, Finland, [μ]University of Oulu, Oulu, Finland, [^]Turku University Hospital, Wellbeing Services County of Southwest Finland, Turku, Finland, [±]Tampere University Hospital, Wellbeing Services County of Pirkanmaa, Tampere, Finland, [¤]Oulu University Hospital, Wellbeing Services County of North Ostrobothia, Oulu, Finland, [§]Finnish Institute for Health and Welfare, Helsinki, Finland.

Georgia/Florida Clinical Center: Richard McIndoe, Ph.D., PI[^4,10], Desmond Schatz*, M.D.*[4,7,8], Diane Hopkins[^11], Michael Haller, M.D.*[13], Melissa Gardiner[^11], Ashok Sharma[^], Ph.D.[^], Laura Jacobsen, M.D.*[13], Percy Gordon[^], Jennifer Hosford*.

[^]Center for Biotechnology and Genomic Medicine, Augusta University, Augusta, GA, USA. *University of Florida, Pediatric Endocrinology, Gainesville, FL, USA.

Germany Clinical Center: Anette G. Ziegler, M.D., PI[±1,3,4,10], Ezio Bonifacio Ph.D.*, Cigdem Sanverdi[±], Anja Heublein[±], Sandra Hummel, Ph.D.[±2], Annette Knopff[±7], Melanie Köger[±], Sibylle Koletzko, M.D.[¶12], Claudia Ramminger[±11], Roswith Roth, Ph.D.[±8], Jennifer Schmidt[±], Marlon Scholz[±], Joanna Stock[±8,11,13], Katharina Warncke, M.D.[±13], Lorena Müller[±], Christiane Winkler, Ph.D.[±2,11].

[±]Forschergruppe Diabetes e.V. and Institute of Diabetes Research, Helmholtz Zentrum München, Forschergruppe Diabetes, and Klinikum rechts der Isar, Technische Universität München, Neuherberg, Germany. *Center for Regenerative Therapies, TU Dresden, Dresden, Germany, [¶]Dr. von Hauner Children's Hospital, Department of Gastroenterology, Ludwig Maximillians University Munich, Munich, Germany.

Sweden Clinical Center: Åke Lernmark, Ph.D., PI[1,3,4,5,6,8,9,10], Daniel Agardh, M.D., Ph.D.[6,12], Carin Andrén Aronsson, Ph.D.[2,11,12], Rasmus Bennet, Corrado Cilio, Ph.D., M.D.[6], Susanne Dahlberg, Malin Goldman Tsubarah, Emelie Ericson-Hallström, Lina Fransson, Emina Halilovic, Susanne Hyberg, Berglind Jonsdottir, M.D., Ph.D.[11], Naghmeh Karimi, Helena Elding Larsson, M.D., Ph.D.[6,13], Markus Lundgren, M.D., Ph.D.[13], Jessica Melin, Ph.D.[11], Kobra Rahmati, Anita Ramelius, Falastin Salami, Ph.D., Anette Sjöberg, Evelyn Tekum Amboh, Carina Törn, Ph.D.[3], Terese Wiktorsson.

Lund University, Lund, Sweden.

Past staff: Eva Andersson, Marie Andersson Turpeinen, Rawya Antar, Maria Ask, Jenny Bremer, Sylvia Bianconi Svensson, Ulla-Marie Carlsson, Magdalena Delikat Kulinski, Annika Fors, Ulla Fält, Thomas Gard, Joanna Gerardsson, Monika Hansen, Anna Hansson, Carina Hansson, Gertie Hansson, Elin M. Hård af Segerstad, Ph.D.[2], Hanna Jisser, Fredrik Johansen, Linda Jonsson, Silvija Jovic, Sigrid Lenrick Forss, Barbro Lernmark, Ph.D.[8], Marielle Lindström, *Maria Markan, Theodosia Massadakis, Marlena Maziarz, Ph.D., Zeliha Mestan, Maria Månsson Martinez, Caroline Nilsson, Emma Nilsson, Yohanna Nordh, Karin Ottosson, Sara Rang, Anna Rosenquist, Monika Sedig Järvirova, Sara Sibthorpe, Birgitta Sjöberg, Ulrika Swartling Ph.D.[8], Erika Trulsson,* Ulrika Ulvenhag, *Anne Wallin, Ingrid Wigheden, Åsa Wimar, Sofie Åberg.*

Washington Clinical Center: William A. Hagopian, M.D., Ph.D., PI[^1,3,4,6,7,10,12,13], Michael Killian[*6,7,11,12], Claire Cowen Crouch[*11,13], Jennifer Skidmore[*2], Ben Kim*, Cody McCall*, Arlene Meyer*, Jared Radtke*, Shreya Roy*.
[^]Indiana University, Indianapolis, IN, USA. *Pacific Northwest Research Institute, Seattle, WA, USA.

Pennsylvania Satellite Center: Dorothy Becker, M.D., Margaret Franciscus, MaryEllen Dalmagro-Elias Smith[2], Ashi Daftary, M.D., Mary Beth Klein, Chrystal Yates.

Children's Hospital of Pittsburgh of UPMC, Pittsburgh, PA, USA.

Data Coordinating Center: Jeffrey P. Krischer, Ph.D., PI[1,4,5,9,10], Rajesh Adusumali, Sarah Austin-Gonzalez, Maryouri Avendano, Sandra Baethke, Brant Burkhardt, Ph.D.[6], Martha Butterworth[2], Nicholas Cadigan, Joanna Clasen, Ph.D., Kevin Counts, Laura Gandolfo, Jennifer Garmeson, Veena Gowda, Shu Liu, Xiang Liu, Ph.D.[2,3,8,13], Kristian Lynch, Ph.D. [6,8], Jamie Malloy, Lazarus Mramba, Ph.D.[2], Cristina McCarthy[11], Hemang M. Parikh, Ph.D.[3,8], Cassandra Remedios, Chris Shaffer, Susan Smith[11], Noah Sulman, Ph.D., Roy Tamura, Ph.D.[1,2,11,12,13], Dena Tewey, Henri Thuma, Michael Toth, Ulla Uusitalo, Ph.D.[2], Kendra Vehik, Ph.D.[4,5,6,8,13], Ponni Vijayakandipan, Melissa Wroble, Jimin Yang, Ph.D., R.D.[2], Kenneth Young, Ph.D.

Past staff: Michael Abbondondolo, Lori Ballard, Rasheedah Brown, David Cuthbertson, Stephen Dankyi, Christopher Eberhard, Steven Fiske, David Hadley, Ph.D., Kathleen Heyman, Belinda Hsiao, Christina Karges, Francisco Perez Laras, Hye-Seung Lee, Ph.D., Qian Li, Ph.D., Colleen Maguire, Wendy McLeod, Aubrie Merrell, Steven Meulemans, Jose Moreno, Ryan Quigley, Laura Smith, Ph.D.

University of South Florida, Tampa, FL, USA.

Autoantibody Reference Laboratories: Liping Yu, M.D.^[5], Dongmei Miao, M.D.^, Kathleen Gillespie*[5], Kyla Chandler*, Olivia Pearce*, Sarah Stollery*, Elinor Balch*, Hanah Batholomew*, Zahra Hashmi*.

^Barbara Davis Center for Childhood Diabetes, University of Colorado Denver, *Bristol Medical School, University of Bristol, UK.

Genetics Laboratory: Stephen S. Rich, Ph.D.[3], Wei-Min Chen, Ph.D.[3], Suna Onengut-Gumuscu, Ph.D.[3], Emily Farber, Rebecca Roche Pickin, Ph.D., Jonathan Davis, Jordan Davis, Dan Gallo, Jessica Bonnie, Paul Campolieto.

Center for Public Health Genomics, University of Virginia, Charlottesville, VA, USA.

HLA Reference Laboratory: William Hagopian[3], M.D., Ph.D., Jared Radtke.

Pacific Northwest Research Institute, Seattle, WA, USA.

(Previously Henry Erlich, Ph.D.[3], Steven J. Mack, Ph.D., Anna Lisa Fear. Center for Genetics, Children's Hospital Oakland Research Institute.)

Repository: Chris Deigan.

NIDDK Biosample Repository at Fisher BioServices, Rockville, MD, USA.

(Previously Ricky Schrock, Polina Malone, Sandra Ke, Niveen Mulholland, Ph.D.)

Project scientist: Beena Akolkar, Ph.D.[1,3,4,5,6,7,9,10].

National Institutes of Diabetes and Digestive and Kidney Diseases, Bethesda, MD, USA.

Other contributors: Thomas Briese, Ph.D.[6] (Columbia University, New York, NY, USA). Todd Brusko, Ph.D.[5] (University of Florida, Gainesville, FL, USA). Teresa Buckner, Ph.D.[2] (University of Northern Colorado, Greeley, CO, USA). Suzanne Bennett Johnson, Ph.D.[8,11] (Florida State University, Tallahassee, FL, USA). Eoin McKinney, Ph.D.[5] (University of Cambridge, Cambridge, UK). Tomi Pastinen, M.D., Ph.D.[5,6] (The Children's Mercy Hospital, Kansas City, MO, USA). Steffen Ullitz Thorsen, M.D., Ph.D.[2] (Department of Clinical Immunology, University of Copenhagen, Copenhagen, Denmark, and Department of Pediatrics and Adolescents, Copenhagen University Hospital, Herlev, Denmark). Eric Triplett, Ph.D.[6] (University of Florida, Gainesville, FL, USA).

Committees:

[1]Ancillary Studies, [2]Diet, [3]Genetics, [4]Human Subjects/Publicity/Publications, [5]Immune Markers, [6]Infectious Agents, [7]Laboratory Implementation, [8]Psychosocial, [9]Quality Assurance, [10]Steering, [11]Study Coordinators, [12]Celiac Disease, [13]Clinical Implementation.
